# Pork Consumption and Seroprevalence of Hepatitis E Virus,Thailand, 2007–2008

**DOI:** 10.3201/eid2009.140418

**Published:** 2014-09

**Authors:** Siriphan Gonwong, Thippawan Chuenchitra, Patchariya Khantapura, Dilara Islam, Narongrid Sirisopana, Carl J. Mason

**Affiliations:** Armed Forces Research Institute of Medical Sciences, Bangkok, Thailand

**Keywords:** Hepatitis E, viruses, seroprevalence, Thailand, young adult

## Abstract

The nationwide seroprevalence of hepatitis E IgG was determined among young men in Thailand. Overall seroprevalence was 14% (95% CI 13%–15%); range by province was 3%–26%. Seroprevalence was lowest in the south, an area predominantly occupied by persons of the Islam religion, whose dietary laws proscribe pork.

Hepatitis E virus (HEV), the etiologic agent of hepatitis E, is a nonenveloped, positive–sense single-stranded RNA virus, ≈7.2 kb in length, of the family *Hepeviridae*. HEV, identified in 1990, was a major cause of what was previously called non-A, non-B hepatitis (*1*,*2*). Hepatitis E occurs worldwide in ≈20 million persons annually, causing≈70,000 deaths (*3*). Clinical signs of hepatitis E are similar to those of other hepatitis virus infections; it is a usually self-limiting illness in healthy persons, who have mild symptoms or asymptomatic disease. Occasionally, fulminant hepatitis develops (*1*–*3*). The overall mortality rate of hepatitis E is 0.5%–4%, but it increases to 20% among pregnant women (*3*).

In Thailand, hepatitis E outbreaks have not been reported; however, sporadic cases have been reported from many areas (*4*). The reported annual incidence of hepatitis E in Thailand ranged 0.05–0.09 per 100,000 persons during 2008–2012; no deaths were reported (*4*). The low incidence of hepatitis E testing in Thailand may underestimate virus exposure among its population (*5*). Hepatitis E IgG provides evidence of individual HEV exposure: titers peaked≈7–8 weeks after infection (*1*). Fourteen years after an outbreak of hepatitis E in Kashmir, 21 of 45 patients (47%) had positive results for IgG anti-HEV by using ELISA (*6*). The hepatitis E IgG seroprevalence range was 9%–23% in studies of small or specific populations in Thailand (*7*–*10*). To identify areas of HEV circulation in the country, we conducted a nationwide hepatitis E seroprevalence study of young men in Thailand.

## The Study

We collected serum specimens after gaining informed consent and permission for future studies as part of HIV-1 surveillance among young men in Thailand who were entering the Royal Thai Army (RTA) during 2007–2008. The RTA uses a lottery system to select ≈60,000 young Thai men at the district level of their family residence for enlistment annually. The men enlisted comprise approximately 10% of young men at the district level in Thailand. Sample sizes were calculated to detect a seroprevalence of ≈50% in each province to within 10% of the true value with 95% confidence. A total of 7,760 stratified randomized samples were chosen on the basis of the reported province of residence of the men before RTA entry.

We measured hepatitis E IgG antibody using a commercial IgG ELISA kit following the manufacturer's instructions (DIA.PRO, Milan, Italy). We tested associations between demographic characteristics and hepatitis E prevalence using the χ^2^ 2-tailed test; p value <0.05 was considered statistically significant. We performed the analyses using SPSS version 12 (SPSS Inc; Chicago, IL, USA).

The study population is described in Table 1. Most of the men were 21 years of age, unmarried, had graduated from middle school, and lived in rural areas. The sample size per province was 69–130 persons. The overall crude seroprevalence of IgG against hepatitis E virus was 14% (95% CI 13%–15%). We generated a spatial distribution map of hepatitis E seroprevalence across Thailand by province of residence using ArcView 8.3 (SPSS Inc) (Figure). In the univariate analysis, seropositivity for hepatitis E IgG was associated with age group and residence region, but not with education level, marital status, or residential area (Tables 1,2). 

**Table1:** Univariate analysis results of demographic variables associated with hepatitis E IgG seroprevalence in young Thai men, 2007–2008.

Demographic characteristics	Study subjects, no. (%)*	Hepatitis E IgG seroprevalence, % (95% CI)
Total	7,760 (100)	14 (13–15)
Age group, y†		
18–20	1,164 (15)	18 (15–20)
21	5,359 (70)	13 (12–14)
22–30	1,150 (15)	14 (12–16)
Marital status		
Single	6,067 (80)	14 (13–15)
Married	1,509 (20)	14 (12–16)
Education level		
Primary school or less	2,121 (27)	14 (12–15)
Middle school	2,641 (34)	14 (13–15)
Senior high school and vocational	1,920 (25)	14 (12–15)
Diploma and high vocational	756 (10)	13 (10–15)
Bachelor’s degree	305 (4)	15 (11–19)
Residential area		
Urban	2,503 (39)	14 (13–16)
Rural	3,896 (61)	13 (12–14)

*Number in each demographic characteristic does not add to the total number of study subjects because of missing data; the number of study subjects with data missing for age group, marital status, education level, and residential area are 87, 184, 17, and 1,361, respectively. Study subjects include all social/religious groups.

**Figure:**
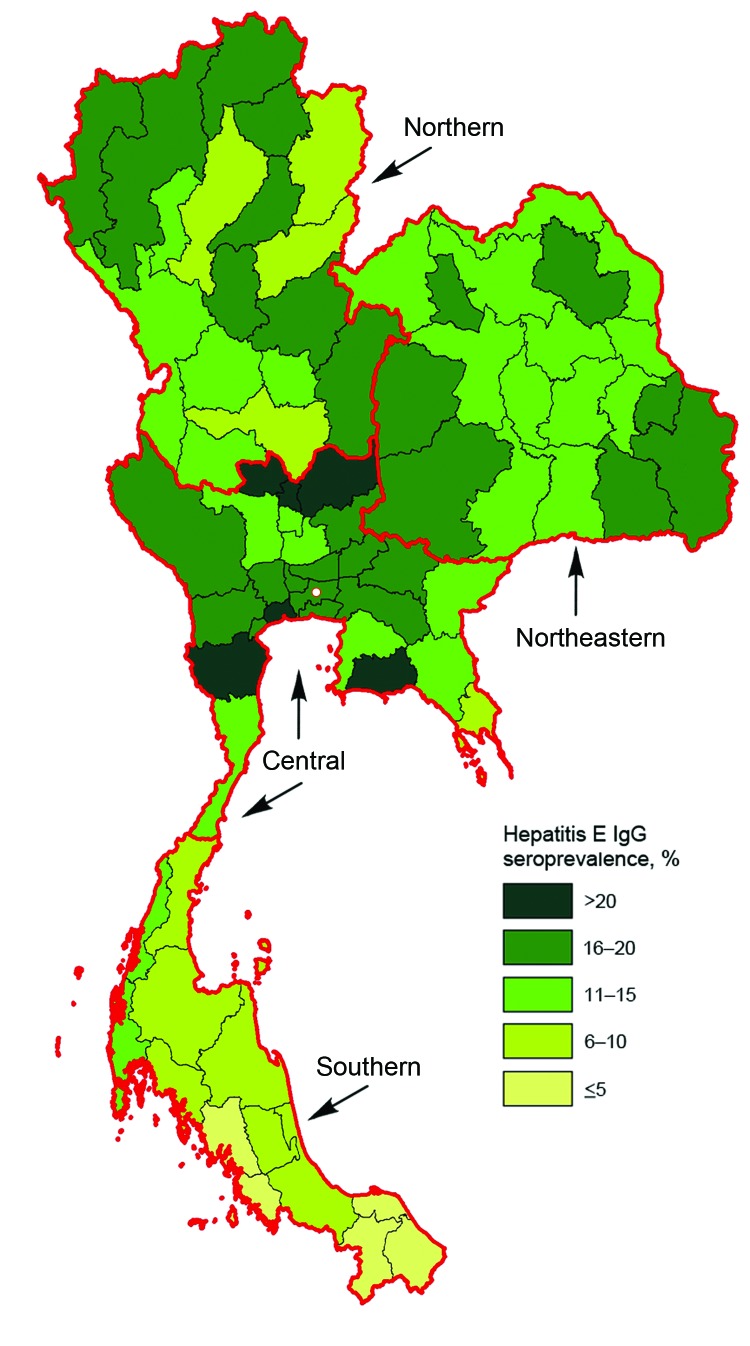
Map of Thailand, showing hepatitis E IgG seroprevalence in young Thai men, 2007–2008, grouped by reported province of residence during the 2 years before entry in to the Royal Thai Army. Circle indicates Bankok, the capital.

**Table2:** Relationship of hepatitis E and pork consumption: hepatitis E IgG seroprevalence by region, and average percentage in each region populated by Muslims, whose Islamic dietary laws proscribe pork consumption, Thailand, 2007–2008

Region of residence*	Hepatitis E IgG seroprevalence, % (95% CI)	Median % Muslim residents (Q1, Q3)†
North	14 (12–15)	0.10 (0.00, 0.20)
Northeast	14 (13–16)	0.10 (0.10, 0.10)
Central	17 (16–19)	0.70 (0.20, 2.80)
South	7 (6–9)	20.15 (10.90, 67.80)
Average	14 (13–15)	0.20 (0.10, 2.67)

*χ^2^ test statistically significant (2-sided p<0.05). †Q1, first quartile; Q3, third quartile.

The hepatitis E IgG seroprevelence was lowest in the southern region. This region has the highest percentage of persons whose religion is Islam, known as Muslims, in Thailand. Because consumption of pork is proscribed by the religion of Islam, the region had the lowest pork consumption and pork production (Table 2) (*11*).

This study determined the nationwide seroprevalence of hepatitis E IgG in Thailand in young men from all 76 provinces. The overall hepatitis E seroprevalence at 14% (95% CI 13%–15%) is similar to results of previous hepatitis E seroprevalence studies in Thailand (9%–23%) (*7*–*10*). However, these comparisons are limited by differences in study design and the performance of the ELISA kits used (*12*). Our study and previous studies used an ELISA to measure antibodies against HEV capsid protein antigens. The range of hepatitis E seroprevalence by province was 3%–26%, indicating HEV exposure of young men across the country. The province of residence was defined as the main province of residence during the 2 years before military service entry. Young men from Thailand typically do not migrate: according to a 2008 national migration report, the percentage of migration between regions by Thai men 20–24 years of age was 8.5% (*13*). Our study population was similar; 10.6% and 9.7% reported a difference between birth and residence at the province and region level, respectively.

HEV is transmitted by the enteric route and is thought to have human and zoonotic reservoirs (*1*–*3*). In Thailand, the route of HEV transmission has not been clearly identified. Studies in Thailand suggested the association of hepatitis E transmission with pork contact and pork consumption (*9*,*10*,*14*). Previous studies noted higher hepatitis E seroprevalence in persons who frequently consume pig organ meat and in swine farmers than in poultry farmers and government officers (*9*,*10*). Moreover, HEV isolated from recovered Thai patients was shown to be closely related to HEV isolated from swine in Thailand (*14*).

## Conclusions

In this study, hepatitis E seroprevalence was not associated with education level, marital status, or type of residence; but was associated with residential region (Table 1) and subsequently, consumption of pork. Hepatitis E seroprevalence level was lowest in the south, especially in the most southern areas where the highest percentage (≥67%) of the non-pork–eating Muslim population resides, such as Yala, Narativat, Satul, and Pattani, which showed a hepatitis E seroprevalences of 4%, 4%, 3%, and 3%, respectively (*11*). These findings support the association of HEV transmission with pork consumption in Thailand (*10*). Given the previously reported differential exposure risk between large-scale farms and medium-sized farms (*15*), it is not unexpected that this study did not find any association between hepatitis E seroprevalence and the number of swine per province (data not shown).

HEV has 1 serotype with 4 major genotypes (G1–G4) (*1*). G1 has been associated with cases in developing countries in Asia and Africa (*1*). G2 was reported in outbreaks in Mexico and West Africa (*2*). G3 was found in sporadic cases from industrialized countries associated with consumption of HEV-contaminated food and is the most prevalent genotype in swine infections worldwide (*2*). The G4 genotype was identified in infected persons and swine in Asia (*1*).

In Thailand, reported swine HEVs belong to the G3 genotype and were genetically closely related to HEV isolates from patients (*14*). HEV G3 may be the main genotype circulating in Thailand. HEV G3 typically induces asymptomatic disease (*1*). This may be a reason for detecting a higher seroprevalence of HEV IgG compared with that of reports of hepatitis E in Thailand. To clarify these findings and identify circulating genotypes, we recommend a nationwide hepatitis E seroprevalence study of the general population to identify areas of HEV circulation.

This study revealed evidence of widespread HEV circulation in Thailand. Hepatitis E is not currently a major public health problem in Thailand, but outbreak reports in many countries in Asia, including the neighboring countries of Vietnam and Myanmar, suggest that outbreak prevention and disease awareness of hepatitis E in Thailand should be enhanced (*2*). Prevention of widespread HEV infection in Thailand may be accomplished by providing information to the general population about proper sanitation and adequate cooking of pork and to the food industry regarding biosafety practices while handling and slaughtering potential reservoir animals.
